# Quantifiable diagnosis of muscular dystrophies and neurogenic atrophies through network analysis

**DOI:** 10.1186/1741-7015-11-77

**Published:** 2013-03-20

**Authors:** Aurora Sáez, Eloy Rivas, Adoración Montero-Sánchez, Carmen Paradas, Begoña Acha, Alberto Pascual, Carmen Serrano, Luis M Escudero

**Affiliations:** 1 Escuela Técnica Superior Ingeniería, Universidad de Sevilla, Seville, Spain; 2 Department of Pathology, Hospital Universitario Virgen del Rocío, Seville, Spain; 3 Instituto de Biomedicina de Sevilla, Hospital Universitario Virgen del Rocío/CSIC/Universidad de Sevilla, Seville, Spain; 4 Neuromuscular Disease Unit, Neurology Department, Hospital Universitario Virgen del Rocío, Seville, Spain; 5 Centro de Investigación Biomédica en Red de Enfermedades Neurodegenerativas (CIBERNED), Madrid, Spain

**Keywords:** Computerized image analysis, Network theory, Neuromuscular disease, Systems biology

## Abstract

**Background:**

The diagnosis of neuromuscular diseases is strongly based on the histological characterization of muscle biopsies. However, this morphological analysis is mostly a subjective process and difficult to quantify. We have tested if network science can provide a novel framework to extract useful information from muscle biopsies, developing a novel method that analyzes muscle samples in an objective, automated, fast and precise manner.

**Methods:**

Our database consisted of 102 muscle biopsy images from 70 individuals (including controls, patients with neurogenic atrophies and patients with muscular dystrophies). We used this to develop a new method, Neuromuscular DIseases Computerized Image Analysis (NDICIA), that uses network science analysis to capture the defining signature of muscle biopsy images. NDICIA characterizes muscle tissues by representing each image as a network, with fibers serving as nodes and fiber contacts as links.

**Results:**

After a ‘training’ phase with control and pathological biopsies, NDICIA was able to quantify the degree of pathology of each sample. We validated our method by comparing NDICIA quantification of the severity of muscular dystrophies with a pathologist’s evaluation of the degree of pathology, resulting in a strong correlation (R = 0.900, *P* <0.00001). Importantly, our approach can be used to quantify new images without the need for prior ‘training’. Therefore, we show that network science analysis captures the useful information contained in muscle biopsies, helping the diagnosis of muscular dystrophies and neurogenic atrophies.

**Conclusions:**

Our novel network analysis approach will serve as a valuable tool for assessing the etiology of muscular dystrophies or neurogenic atrophies, and has the potential to quantify treatment outcomes in preclinical and clinical trials.

## Background

Neuromuscular diseases (NMD) comprise a large and heterogeneous group of disorders affecting the motor unit [1-3]. NMD can encompass acquired and genetic etiologies with very diverse clinical, morphological and molecular characteristics that usually (but not always) manifest in a progressive manner [2,4]. A comprehensive analysis of a muscle biopsy is necessary for a complete and detailed evaluation of a NMD [5]. However, the morphological analysis of muscle samples is mostly a subjective process and difficult to quantify.

**Figure 1:**
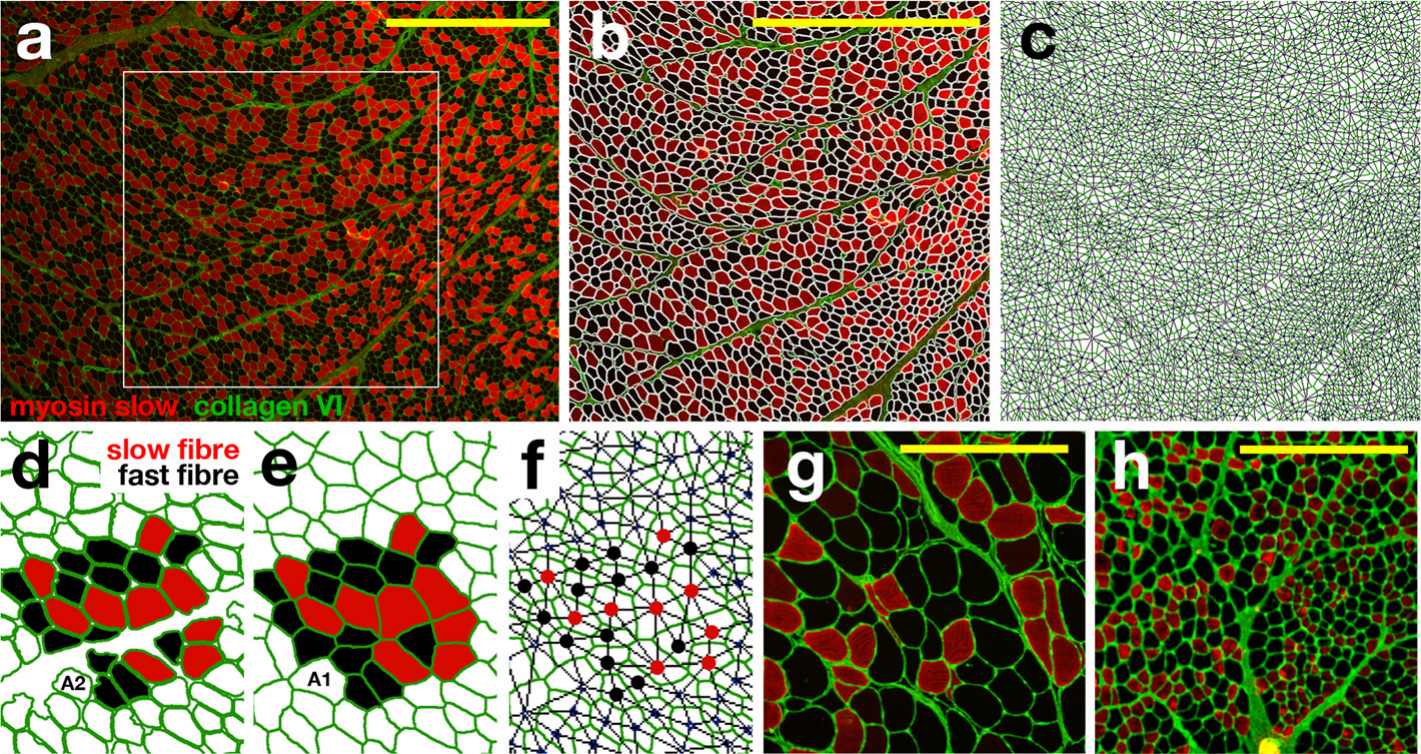
**Segmentation and network construction of a muscle biopsy image. (a)** Fluorescence image corresponding to a control biopsy showing collagen VI content including the endomysium and perimysium (green), slow fibers (red) and fast fibers (black). The white square delimits the region of interest used in this case. **(b)** Segmented image enabling all fibers to be identified. The fibers are considered objects (white boundary lines) whose geometric properties can be calculated. **(c)** This information is used to produce a muscle network where each fiber is represented as a node, and two nodes are connected if two fibers are adjacent in the muscle biopsy. **(d)** Detail of the segmented image showing objects corresponding to the fibers (green boundary lines). The area of each object is termed A2 for subsequent calculations of the index of fibrosis. NDICIA identifies ‘slow’ and ‘fast’ fibers shown in red and black, respectively. **(e)** The objects are expanded in a linear manner until they reach the adjacent objects. This allows each fiber’s neighbors to be identified. A1 is the area of the expansion for each object, and is also used for calculation of the fibrosis index for each image. **(f)** Detail of the network corresponding to the region in (e). The network is formed by slow and fast nodes shown as red and black dots, respectively. **(g,h)** Region of interest selected from NA (BNA01-1, g) and MD (QD54-1, h) biopsies analyzed in this study. The collagen VI content (green), slow fibers (red) and fast fibers (black) are marked. Scale bar, 500 mm.

Skeletal muscle fibers are bundled together in fascicles that are separated by the perimysium, a connective tissue sheath that conveys innervation and vascular irrigation. Each muscle fiber is in turn enclosed by a very thin band of connective tissue, the endomysium. Skeletal muscles fibers can be classified into type I (slow) or type II (fast) fibers, which are distributed in a mosaic pattern along the fascicles [6,7]. A transverse section of a normal muscle shows the muscle fibers with a polygonal shape and homogeneous size, surrounded by a thin mesh of collagen (Figure 1a). NMD can be classified as neurogenic disorders, myopathies and neuromuscular junction disorders [1-3]. Muscular dystrophies (MD) are primary myopathies characterized by a wide variation in fiber size, a rounded shape of atrophic fibers, and fibrosis (an increase of endomysial collagen), which define the dystrophic pattern. In neurogenic atrophies (NA), motor neurons or peripheral nerves are primarily affected. Groups of angulated atrophic fibers, together with fascicular atrophy and fiber-type grouping secondary to reinnervation events are seen. Muscles also can show a combination of neurogenic and myopathic patterns because additional myopathic features or fibrosis may be present in chronic neuropathic processes [2,8]. Therefore, the correct interpretation of a condition based on its pathologic features cannot always be determined with certainty, thus hampering the diagnosis of the underlying disease. A large panel of different histochemical and histoenzymatic techniques are necessary to identify pathologic changes in the routine diagnostic process [9,10] (Additional file 1: Figure S1). Previous attempts to automate the extraction of geometrical characteristics from normal muscle biopsies have been published [11-16], but those methods fail to provide an automated analysis or adequate scrutiny of the information derived from the analysis. Our analysis begins at this point, taking into account a large number of samples to study both geometrical and network data to include morphometric and organizational information.

Network science involves the study of the generally asymmetric relationships that exist between elements that form complex systems. This approach has been used successfully to capture information from systems ranging from the Internet to social networks [17-20]. Although network analysis has been recently exploited to investigate biological systems of different scales [21], the application of network theory at the level of individual cells or groups of cells has been relatively limited [22-24]. A recent report introduced network theory to the study of tissue organization [22]. In that report, the approach followed allowed the organization of epithelial networks to be described objectively. Interestingly, the method permitted the quantification of differences between wild-type and mutant (perturbed) tissues. Muscle biopsies share a similar structure with epithelia due to the polygonal appearance of the fibers. However, muscle samples present extra heterogeneity due to the presence of endo- and perimysium together with the different fiber types. Our novel method, named Neuromuscular DIseases Computerized Image Analysis (NDICIA), identifies differences between normal and affected muscles in an objective, reproducible and precise way. The application of NDICIA to the study of muscle biopsies could have clinical application as a new diagnostic tool that would significantly aid in the evaluation of the degree of pathology and the collection of data in both clinical practice and in the research laboratory.

## Methods

### Tissue sampling

For the retrospective analysis of human muscle tissue, we obtained images from processed biopsies stored in tissue banks at the Virgen del Rocío University Hospital (Seville), the Santa Creu i Sant Pau Hospital (Barcelona) and the Doce de Octubre Hospital (Madrid). Our database consisted of 102 images from 70 individuals. Fifty images were considered controls and came from patients who were referred to the Neuromuscular Unit of Virgen del Rocío University Hospital because of minor symptoms, such as unspecific fatigue or pain, in whom the physical examination and complete routine biopsy analysis were completely normal. Seventeen images came from adults with diverse NA and 20 images were from the quadriceps of children with either Duchenne or Becker MD (Additional file 2: Table S1). All the methodology used in this study follows the guidelines of the Declaration of Helsinki developed by the World Medical Association. The biopsies used in this study were treated following the requirements established by Spanish law in relation to the Investigation in Biomedicine (Ley 14/2007), personal data protection (Ley Orgánica de 15/1999) and Bioethics. The Hospital Virgen del Rocío ethics commission gave approval for this work (File 2/11). All biopsies were performed under informed consent using a standardized protocol [10].

### Immunohistochemistry

Muscle biopsies were processed by the standard methods of freezing and cryostat sectioning. Fluorescence microscopy was used to detect muscle fiber types and the amount of collagen in preparations. The following antibodies were used in accordance with standard immunostaining protocols [10]: mouse anti-myosin heavy chain (slow) (Leica, Newcastle, United Kingdom, clone WB-MHCs; 1:200), mouse anti-myosin heavy chain (fast) (Leica, Newcastle, United Kingdom, clone WB-MHCf; 1:200), and rabbit anti-collagen type VI (Millipore, Temecula, CA, USA, lot number: NG18332|0; 1:300). After staining, the preparations were mounted using Fluoromount-G (Southern Biotech, Birmingham, AL, USA All images were obtained using the 10× objective of a DP70 Olympus microscope (Olympus Iberia, Barcelona, Spain), with a resolution of 4080 × 3072 pixels. Regions of interest were chosen that did not show artifacts caused by the sample processing protocol.

### Biopsy evaluation

The pathologist from the Virgen del Rocío University Hospital estimated the degree of severity (from 1 to 4) of the MD samples by following routine and specific protocols (see Additional file 1: Figure S1).

### Image processing: muscle fiber segmentation

Muscle fiber segmentation for image processing was carried out in two steps. First, muscle fibers in an image were identified by applying morphological operators. This was followed by application of a watershed transform to provide accurate detection of the fiber contours.

The G component of the Red-Green-Blue (RGB) image was used because of the high contrast that exists between muscle fibers and collagen. Considering that fibers are darker than the surrounding collagen, we searched the image for intensity valleys. Accordingly, the h-minima transform [25] was applied to the G component image to obtain homogeneous minima valleys. The h-minima or h-maxima transform is a powerful mathematical tool used to suppress undesired minima or maxima [25]. By using the h-minima transform, all minima whose depth was lower than or equal to a given h-value were suppressed. The h-value has a direct influence on the number of segmented regions: the larger the h-value is, the smaller the number of segmented regions. In our case, the h-value was experimentally chosen as being half the average intensity of G, such that:

*ℎ =* ed transform may be applied to an original image as given. However, if the gradient of the original image is used, the minima in the gradient image will correspond to sites within homogeneous regions in the original image. Thus, the watershed transform is usually applied to the gradient image. The watershed algorithm yields results with substantial over-segmentation; that is, the number of segmented regions could be much larger than desired, with clearly identifiable objects or regions being broken into multiple smaller regions. This undesirable result is because the gradient image used in the process is sensitive to noise. The problem of over-segmentation can be overcome with the use of markers that identify objects. The objects’ contours in the gradient image can be seen as the highest crest-lines around the object marker. In our case, the image gradient was calculated in the G component and the internal and external markers were derived from the first step. The block diagram of Additional file 3: Figure S2 shows the steps followed in the segmentation process.

### Image processing: generation of the muscle network

For a structural analysis of the biopsy images, we formed a muscle network, where each fiber is represented as a node and two nodes are connected if two fibers are adjacent. This network was formed from the external markers, that is, from the watershed transformation directly to the binary image considered as internal markers, resulting in the first step of the segmentation process. The resulting image was a mosaic where each fiber contour reached the adjacent fiber contour.

### Geometric and network feature extraction

The next step concerned feature extraction. Geometric features such as the fiber area or the length of the major and minor axes of the fiber were extracted from the detected contours. Other parameters that took into account the neighboring vicinity of each fiber, such as the ratio between the fiber area and adjacent fiber areas, or the ratio between the fiber area and the area resulting from the expansion of its contour (computed in the previous step), were calculated from the muscle network. Finally, graph theory was applied to the muscle network to compute additional features.

A total of 82 characteristics were computed, including 16 geometric features, 22 features derived from the muscle network, and 44 from graph theory (Additional file 4: Table S2).

### Discriminant feature selection

A feature selection step was performed to analyze the discriminatory power of the 82 characteristics mentioned above. To achieve this, the sequential forward selection method (SFS) [26] and the sequential backward selection method (SBS) [26] used via the Fuzzy-ARTMAP neural network [27] were applied.

SFS is a bottom-up search procedure where one feature at a time is added to the current feature set. At each stage, the feature to be included in the feature set is selected from among the remaining available features, which have not been added to the feature set. The new enlarged feature set yields a minimum classification error compared to adding any other single feature. The algorithm stops when the addition of a new feature yields an increase of the classification error. The SBS is the top-down counterpart of the SFS method. It starts from the complete set of features and, at each stage, the feature that shows the least discriminatory power is discarded. The algorithm stops when removing another feature results in an increase of the classification error.

The selection performance was evaluated by five-fold cross-validation [26]. In this way, the disadvantage of sensitivity to the order of presentation of the training set that the SBS and SFS methods present was diminished. To perform the cross-validation method, four disjoint subsets of each class (control, dystrophy) were used. Three of these subsets served as a training set for the neural network, and the remaining one was used as a validation set. The procedure was then repeated, interchanging the validation subset with one of the training subsets and so on until each of the four subsets had been used as validation sets. The final classification error was calculated as the mean of the errors for each cross-validation run.

The classifier used was the Fuzzy-ARTMAP neural network architecture developed by Carpenter *et al.*[27], which is based on adaptive resonance theory (ART). Fuzzy-ARTMAP is a supervised learning classification architecture for analog-value input pairs of patterns where each individual input is mapped to a class label.

### Results analysis

Once the feature vector with the most discriminatory power to distinguish between the classes analyzed had been selected, it was possible to classify new biopsy images and to estimate the degree of pathology with respect to control images.

**Figure 2:**
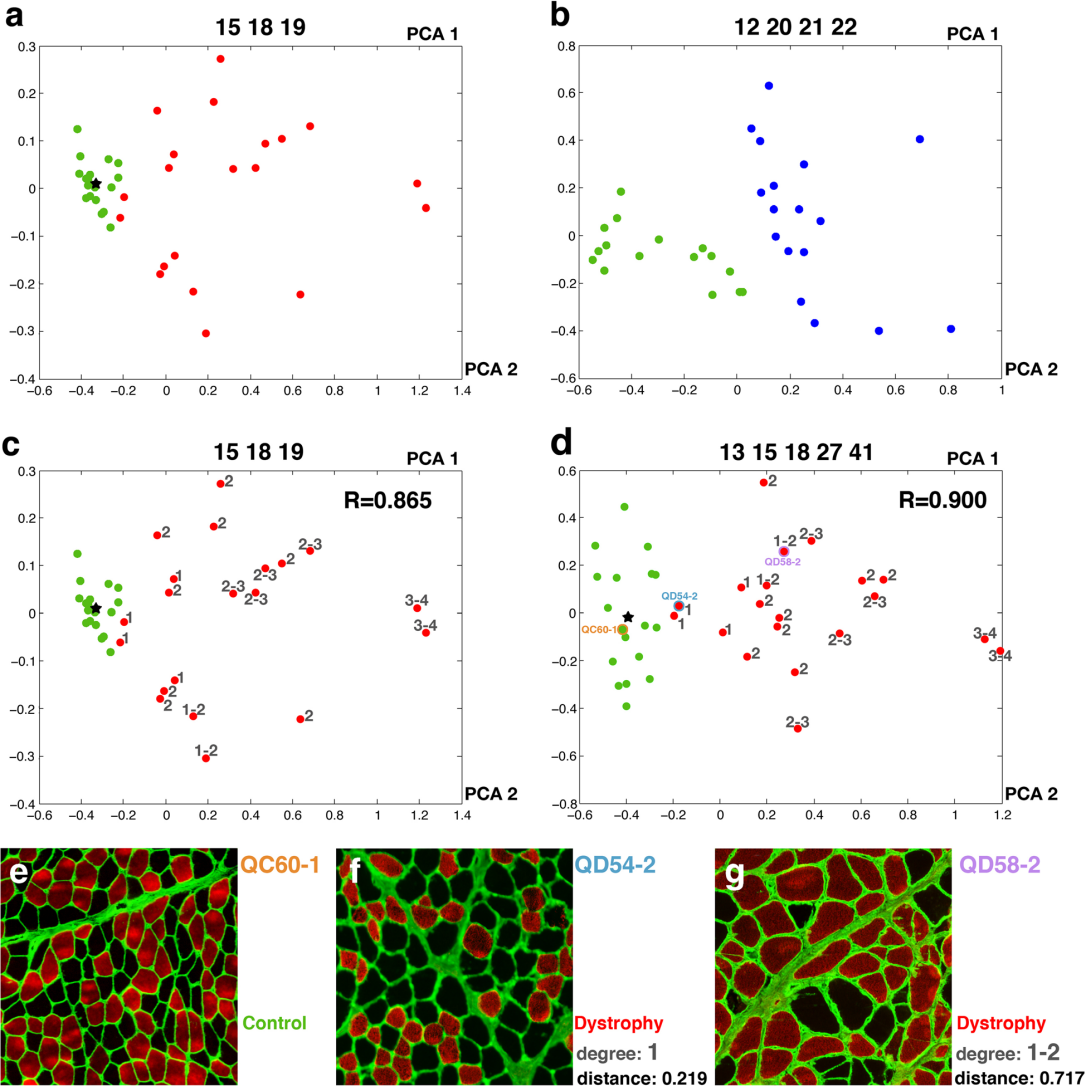
**Principal component analysis graphs of the comparisons of different control and pathological samples.** PCA graphs for the comparisons of muscle images from different sources using different groups of characteristics. The green dots represent the control images (quadricep biopsies from children in a, c, and d and adult biceps in b). The black star represents the centroid for the control dataset in each graph. The red and blue dots are the MD and NA images, respectively. **(a)** Control versus MD comparison using characteristics 15, 18 and 19. **(b)** Control versus NA comparison using characteristics 12, 20, 21 and 22. **(c)** Same comparison as in (a), showing the degree of pathology as evaluated by the pathologist and the correlation coefficient with distances to the centroid. **(d)** Control versus MD comparison using characteristics 13, 15, 18, 27 and 41 and showing the degree of pathology and the correlation with distances to the centroid. Three images (dots) are highlighted with an orange, light blue or violet circle. These dots correspond to the images in e, f and g respectively. **(e)** Detail of a representative control image (the one closer to the centroid, QC60-1). **(f)** Detail of the QD54-2 image. This image shows a small increase in the amount of collagen between the fibers and in the heterogeneity of sizes and shapes of them. **(g)** Detail of the QD58-2 image. This image presents a clear increase of the endomysium and higher heterogeneity in sizes and shapes than in e and f. PCA, principal component analysis.

The Fuzzy-ARTMAP neural network classifies new biopsies based on the selected features. Also, principal component analysis (PCA) [26] allows the degree of pathology of each biopsy image to be visualized graphically based on its position when two or three principal components are represented (Figure 2). These principal components are correlated data points of the feature vector that PCA transforms into a small number of uncorrelated variables. The projection maximizes the dispersion of the individual data points without prior knowledge of whether these are expected to form groups. This allows the unbiased identification of naturally separated sets of data points that may form groups because they are similar.

PCA was applied to the selected feature vector. To check the vector’s performance as an indicator of the degree of pathology, the Pearson correlation coefficient was computed. This parameter provides a measure of the strength of linear dependence between two variables X and Y, giving a value between +1 and -1 inclusive. In this case, the variable X is the degree of pathology evident in the biopsies used in the training stage, which have previously been analyzed by the pathologist. The variable Y is the Euclidean distance between the pathological images and the centroid of the controls in the PCA graph.

## Results

### NDICIA identifies fibers in muscle samples and extracts the muscle network

Immunohistochemical staining of the collagen network surrounding the muscle fibers (endomysium and perimysium) with anti-collagen VI antibody enabled quantification of the amount of collagen in the tissue and, at the same time, provided an outline of the muscle fibers. The muscle fiber type was identified using anti-myosin slow (type I) or anti-myosin fast (type II) specific antibodies (Figure 1a). By applying a digital image processing algorithm based on the watershed transform [28], the segmentation process identified cell contours and enabled geometrical characteristics such as area or shape indicators to be identified, as well as the muscle fiber type (fast or slow) to be determined (Figure 1b,d and Methods). The segmented image also served as the basis for determining which cells were adjacent to each fiber (Figure 1f). This information was used to produce a muscle network where each fiber was represented as a node, and two nodes were connected if two fibers were situated adjacently (Figure 1e,f). This approach was used to extract typical characteristics of the three types of images: control, NA and MD (Figure 1a,g and h respectively).

### NDICIA selects the most discriminating features for the optimal classification of muscle samples

The network and segmented images were used to extract 82 characteristics from muscle biopsies (Additional file 4: Table S2). Sixteen of these characteristics were related to geometrical features of the biopsy and reflected the basic features analyzed by the pathologist. Aiming to capture the organization of the fibers in the muscle, we added eight characteristics that were exclusively related to the structure of the network formed by the tissue: the number of neighbors (characteristics 17 and 18), the number of neighbors for a particular type of fiber (19 and 20), and the number of neighbors of one kind for a particular type of fiber (21 to 24). A further 14 characteristics (25 to 38) were obtained by modifying the geometric parameters to convert them into network characteristics. The remaining 44 network features consisted of inherent network properties that would not normally form part of the evaluation by the pathologist (described in the legend of Additional file 4: Table S2). The combination of these 82 geometrical and network characteristics gives rise to a ‘Feature vector’ that describes the properties of the image. This allows the application of multivariate statistical analysis using PCA [19,29,30]. To evaluate the discriminatory power of each individual feature to classify and select the most effective subset of features, we used the SFS and SBS methods [27] via an artificial neuronal network (see Methods and Additional file 5: Figure S3).

First, we tried to mimic the routine analysis made by the pathologist using only the first 24 characteristics of our set of 82 characteristics. The selection of relevant features for each comparison required a training process based on known biological data. Performing the training by using 18 images from control and 20 images from MD samples (Additional file 2: Table S1), NDICIA selected one geometrical (15) and two network characteristics (18 and 19) for the identification of dystrophies. PCA graphs for the control and MD datasets were generated using the selected features. Whereas the control images were clustered at one side of the projection, the MD dataset was separated from the control group and more spread out (Figure 2a).

We repeated the analysis with other sets of images that could be considered more similar based on simple visual inspection. We trained NDICIA using 16 control images from adult biceps brachii and 17 NA images from different muscles (Additional file 2: Table S1). The PCA graph also revealed a separation of the images into two groups (Figure 2b). Interestingly, none of the previous characteristics (15, 18, 19) were selected when control and NA samples (12, 20, 21, 22) were compared, thus indicating the flexibility of the program to adapt to different sets of images and pathologies. The three network features selected for NA were related to the organization of the fiber types and were able to capture the NA characteristic fiber-type grouping due to secondary reinnervation events.

### NDICIA objectively quantifies the severity of pathology of muscular dystrophies

PCA graphs can be used to quantify differences between components in different image datasets [22]. We postulated that the distribution patterns in the PCA graphs of MD images with respect to the group of control images could have diagnostic significance, and tested this hypothesis by performing a blind experiment. The pathologist estimated the degree of severity of the MD samples by analyzing the biopsies using the routine panel and the specific protocols of histochemical, histoenzymatic and immunohistochemical techniques (Methods and Additional file 1: Figure S1). We then compared his evaluations with the results of PCA plots generated using the characteristics 1 to 24. Interestingly, we found a high correlation (R = 0.865, *P* <0.0001) between the degree of pathology diagnosed by the pathologist and the Euclidean distance between the MD image and the centroid of the controls in the PCA graph (Figure 2c).

Once we confirmed that the results obtained using 24 characteristics correlated with the pathologist’s evaluation, we decided explore the whole set of 82 available characteristics, looking for novel insights from the network analysis and an improvement of our results. After testing different options, the combination of characteristics 13, 15, 18, 27 and 41 resulted in the maximum correlation with the pathologist’s evaluation (R = 0.900, *P* <0.0001; Figure 2d). Changes in some of these characteristics could be observed in the dystrophic images, such the increase of collagen VI staining or the enhancement in heterogeneity of sizes and shapes of the fibers (Figure 2e-g). By contrast, some network characteristics were too complex to be detected by simple visual inspection. This was the case for ‘standard deviation of the degree of neighbors’ (18) or ‘average strength of fast cells’ (41).

### Classification of muscle biopsies when multiple categories were compared

In practice, pathologists receive new muscle biopsies for analysis that are accompanied by some additional clinical information that orients the evaluation. We simulated this situation, testing if our method was able to correctly classify new images into any our three patterns of interest (control, neurogenic and dystrophic). First, we performed a new feature selection step using more control images to cover a wider range of age and muscle types (87 images: 50 controls, 20 MD, 17 NA; Additional file 2: Table S1). When the training procedure was performed on the 82 features, nine characteristics were subsequently selected (15, 16, 25, 29, 30, 32, 34, 62, 69). It is of interest that only two of these are geometric characteristics, pointing again to the high discrimination power of network features. Then, the pathologist evaluated the morphological pattern (normal, dystrophic or neurogenic) of nine new biopsies that were not used in the different feature selection steps (Table 1). For each image we performed a triple (50 control versus MD versus NA) and two double (control child quadriceps versus MD and control adult biceps versus NA) comparisons. The classification of the four controls and the sample showing a clear dystrophic pattern (X01) was correct. In addition, NDICIA detected a mixed pattern of a dystrophic myopathy with a predominance of type I fibers (X13) and an end-stage NA with associated fibrosis (X05). Case X04 showing slight-moderate NA was considered normal in the triple comparison, but was correctly classified in the control versus NA evaluation, showing that the double comparison step is more sensitive. Finally, case X02 with initial stage NA, which only showed some fiber-type grouping of fibers without atrophy, was classified in both comparisons as a control (Table 1).

**Table 1:** New dataset of images and classification of the different comparisons

**Sample**	**Age (years)**	**Muscle**	**Morphological pattern evaluation**	**ALL 82cc**	**Control versus MD 82cc**	**Control versus NA 82cc**
				**cc = 15, 16, 25, 29, 30, 32, 34, 62, 69**	**cc = 13, 18, 15, 27, 41**	**cc = 21, 33, 55**
X03-1	45	Biceps	Normal	C	No D	No N
X06-1	18	Biceps	Normal	C	No D	No N
X11-1	12	Quadriceps	Normal	C	No D	No N
X12-1	6	Quadriceps	Normal	C	No D	No N
X01-1	55	Biceps	Dystrophic pattern	D	D	No N
X01-2	55	Biceps	Dystrophic pattern	D	D	No N
X01-3	55	Biceps	Dystrophic pattern	D	D	No N
X01-4	55	Biceps	Dystrophic pattern	D	D	No N
X01-5	55	Biceps	Dystrophic pattern	D	D	No N
X13-1	3 months	Quadriceps	Dystrophic pattern	D	D	N
X05-1	54	Gastrocnemius	NA-severe-pseudodystrophy	N	D	N
X02-1	37	Gastrocnemius	NA, only focal grouping	**C**	No D	**No N**
X02-2	37	Gastrocnemius	NA, only focal grouping	**C**	No D	**No N**
X04-1	47	Gastrocnemius	NA, slight-moderate	**C**	No D	N
X04-2	47	Gastrocnemius	NA, slight-moderate	**C**	No D	N

Results of the classification of the three comparisons performed on different test samples. In these comparisons 82 characteristics were used in the feature selection step. The table shows the selected characteristics in each case. Cases where NDICIA was not able to classify the condition correctly are labeled in bold. ‘No D’ corresponds to cases where NA was classified as ‘not dystrophic’, and ‘No N’ the cases with fibrosis was classified as being ‘not neurogenic’ since there were only two options in these double comparisons. C*,* normal pattern, D, dystrophic pattern, N, neurogenic pattern.

### NDICIA can quantify new samples with dystrophic and neurogenic patterns

**Figure 3:**
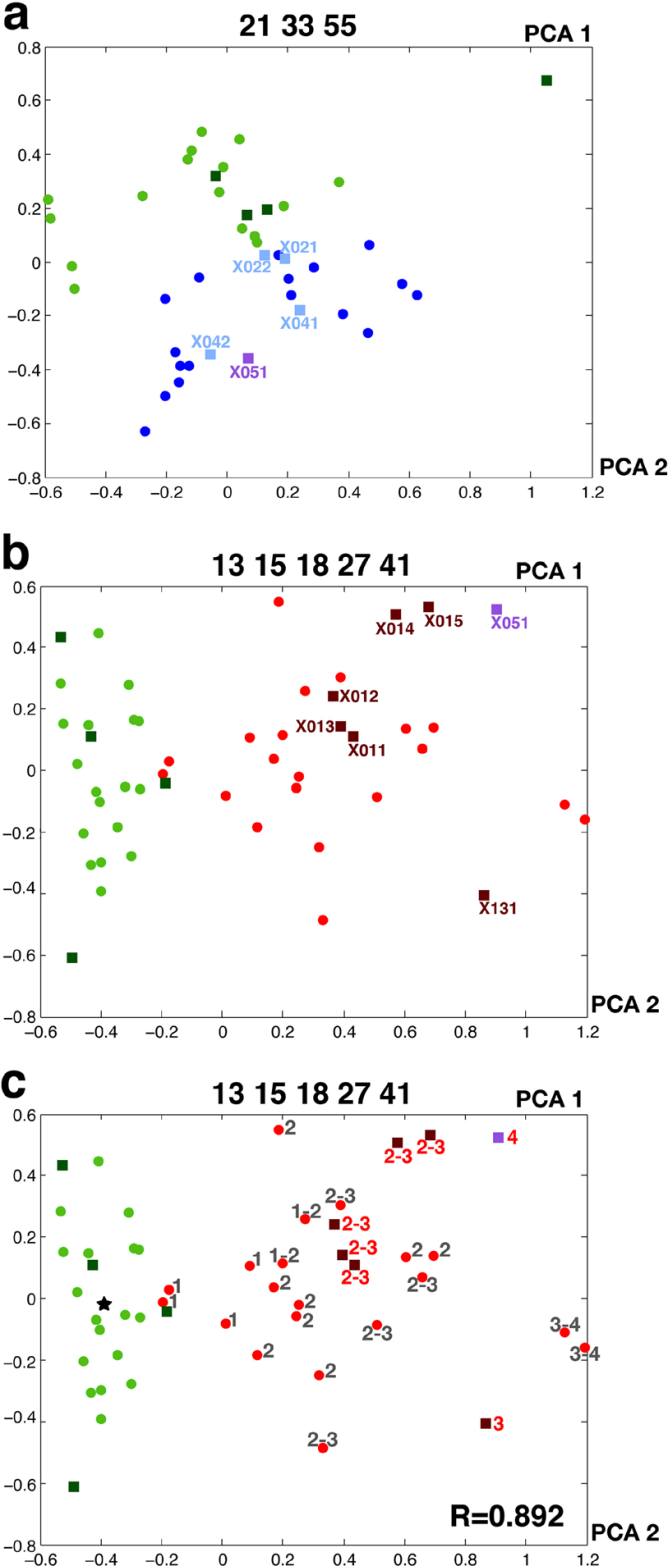
**Principal component analysis graphs of the comparisons of control dystrophies with newly incorporated samples.** (**a**) PCA graphs for the control versus NA comparison using characteristics 21, 33 and 55 (control biceps: green dots; NA: blue dots) incorporating the control (dark green squares) and NA images (light blue squares) from a new dataset. The violet square represents the position of sample X05-1, a mixed neurogenic-dystrophic pattern. (**b**) PCA graphs for the comparisons of muscle images from control quadriceps (green dots) and MD quadriceps (red dots) using characteristics 13, 15, 18, 27 and 41. The position of the new images is represented by dark green squares (new control) and brown squares (new cases with dystrophic pattern). The violet square corresponds to the position of the sample with severe NA affected with dystrophy (X05-1). (**c**) Same plot showing the degree of pathology of the new samples (red numbers) and the correlation with distances to the centroid (black star). PCA, principal component analysis.

We tested if our method could also quantify the severity of these newly incorporated samples. A PCA graph was generated for the control versus NA comparison that included corresponding images from the new set (Figure 3a). The new controls appeared close to the original control samples, whereas the neurogenic images appeared scattered in different positions within the cloud of NA images. Interestingly, X02-1 and X02-2 were located at the boundary between the two groups, indicating the low level of pathology that this patient presented. Similar results were obtained in the case of the control versus MD comparison (Figure 3b). The particular case of X05-1 was assessed in both comparisons and exhibited dual properties that identified it as a neurogenic pathology with a strong fibrosis component (similar to the morphological pattern described by the pathologist). Finally, the evaluation carried out by the pathologist was compared with the NDICIA analysis of these new samples with respect to their distance to the centroid of the controls in the PCA graph (Figure 3c). Importantly, the value of the correlation after the inclusion of the new images remained very high (R = 0.892*, P* <0.0001; Figure 3c), suggesting that NDICIA maintained its quantification power with samples that were not subjected to the feature selection step.

## Discussion

We have described here NDICIA, a flexible method to aid in the diagnosis of different muscle diseases. Our results were based on 87 images from 61 individuals (controls and patients). To the best of our knowledge, this is the largest and most comprehensive dataset of images analyzed in a neuromuscular study. Previous reports have been published of attempts to facilitate the automated extraction of geometric characteristics from muscle biopsies [11-16]. These studies rely on the development of segmentation methods using a very small number of samples to only extract morphometric information. In our case, the segmentation of the muscle images (control and pathological) is only the first step undertaken. NDICIA also extracts topological information, capturing the spatial organization of slow and fast fibers by constructing a ‘muscle network’ of fiber-to-fiber contacts. NDICIA automatically identifies muscle cells and classifies them as type I or type II, calculates the collagen content of the biopsy, and provides values for the geometric and network characteristics of the muscle fibers. It then combines these data to complete a quantifiable characterization of the muscle sample in a reproducible, objective and automated manner. Therefore, the framework introduced here complements, facilitates and accelerates the routine work of the pathologist. NDICIA is able to distinguish between three patterns (control, dystrophic and neurogenic) and point out when a sample presents a mixture of dystrophic and neurogenic patterns. Of course, morphological information needs additional data such as clinical, biochemical or molecular information to reach the final diagnosis, but the useful information provided by NDICIA will doubtless aid the pathologist’s evaluation. As a proof of principle, we demonstrated that the information extracted from the images is meaningful because the quantification provided correlated highly with the pathologist’s evaluation in the case of MD. Besides this practical accomplishment, our results suggest that network science technology can be used to capture defining signatures of muscle biopsies. We assessed the two morphological patterns, MD and NA, to evaluate the accuracy of this method. It is out of the scope of the present work to analyze every type of NMD. However, NDICIA is a flexible tool and we think that it can be very helpful in other groups of muscle disorders in the near future. For example, congenital myopathies share some common morphological features, such as type I fiber atrophy or type I fiber predominance or uniformity, that can be easily recognized by the NDICIA network approach.

We have shown that the characteristics chosen in the feature selection step were different for MD and NA. Using 24 characteristics, NDICIA selected one geometrical (‘A1/A2 relation’) and two network (‘average degree of neighbors’ and ‘standard deviation of the degree of neighbors’) characteristics for the identification of dystrophies. The selection of ‘A1/A2 relation’ is logical because it reflects the level of fibrosis in the biopsy, this being an important biomarker for dystrophies. However, it was surprising that the ‘degree of neighbors’ was more important than features related to the shape of the fibers. Interestingly, none of the previous characteristics (15, 18, 19) were selected when control and NA (12, 20, 21, 22) were compared. The three network characteristics selected are related to the organization of the fiber types and were able to capture the characteristic fiber-type grouping due to secondary reinnervation events. We also obtained different selected features from the analysis of the 82 characteristics (characteristics 21, 33 and 55 for NA; and 13, 15, 18, 27 and 41 for MD). Therefore, NDICIA selects different features that capture the specific changes that occur within a particular NMD. This modular nature of NDICIA establishes an ideal framework to be adapted to the study of any NMD. In some cases, the selection of characteristics from among the 82 that we presented here will be sufficient, whereas in others the addition of new markers to a similar underlying method will be required.

The potential of NCIDIA resides also in the quantification of severity of pathologic pattern. Whereas the first 24 characteristics were designed to ‘imitate’ the parameters that the pathologist typically analyses in a biopsy, the remaining 58 network features are extremely difficult (or even impossible) to evaluate by visual inspection. Our results demonstrated that the inclusion of these network parameters first improves the quantification of the degree of pathology by dystrophies (from R = 0.865 to R = 0.900) and, second, uncovered unexpected network traits that defined the differences between control and MD patterns. These results convincingly point to the average relation neighbors major axis (27) and average strength of fast cells (41) as two new parameters important for the evaluation of MD. The first reflects the homogeneity of fiber shapes and sizes along the image, and the second is an index of fiber packing. We conclude that network characteristics could serve as a valuable tool for assessing the etiology and/or progression of a disease, and may help to further our understanding of the pathological processes that accompany NMD.

We have shown here that NDICIA (at least for MD and NA) was able to quantify the differences between control and pathological samples that were not used in the feature selection step. Remarkably, the results maintained a high degree of correlation with the evaluation of the pathologist (Figure 3c). Although the final diagnosis of some NMD does not require very detailed analysis of morphological characteristics, the precise quantification leads to the identification of subtle differences between our controls and pathological images. For example, the images X02-1 and X02-2 (from a patient with initial stage NA whose sample only showed some focal grouping of fibers without atrophy) were classified as control in the triple and double comparisons (Table 1). Interestingly, when the PCA graphs were analyzed (Figure 3a), we observed that these two images were at the interface of control and NA datasets. We interpret this type of result as an indication that involvement of a possible affectation should be suspected.

## Conclusions

NDICIA´s major achievement is its potential clinical application. The combination of the precise quantification and the incorporation of new samples could allow fine-tuning of the diagnosis of the pathologies analyzed here. This enables the identification of the first signs of pathology, bringing forward the onset of potential treatment. In the neuromuscular field, objectivity in the quantification of outcome measurements is a clear, fundamental demand [31,32]. In addition, the modular nature of NDICIA gives it the potential to be extended to the analysis of any NMD, using new stainings and characteristics. NDICIA fulfills all the requirements for monitoring the progression of a disease in natural history studies and following the treatment results in preclinical and clinical trials. The results presented here are also likely to be applicable to the analysis of complex cellular ensembles associated with other biomedical conditions.

## Abbreviations

ART: adaptive resonance theory; MD: muscular dystrophies; NA: neurogenic atrophies; NMD: neuromuscular diseases; NDICIA: Neuromuscular DIseases Computerized Image Analysis; PCA: principal component analysis; SBS: sequential backward selection; SFS: sequential forward selection

## Competing interests

The authors declare that they have already registered a patent with the content of the method. We declare no further competing interest.

## Authors’ contributions

LME conceived the study with help from AP. ER evaluated and selected the muscle biopsies with help from CP. LME and AM obtained the images. AS processed the images, wrote the software, generated the network and performed all the comparisons and statistical analyses under the supervision and help of BA and CS. All authors participated in the interpretation of results, discussion and the development of the project at all stages. ER, CP, AP, AS and LME wrote the manuscript with input from all authors. All authors read and approved the final manuscript.

## Pre-publication history

The pre-publication history for this paper can be accessed here:

http://www.biomedcentral.com/1741-7015/11/77/prepub

## Supplementary Material

Additional file 1: Figure S1 Procedure for analysis of muscle biopsies in the neuropathology laboratory. Muscle biopsies are processed by cryostat. A large number of slides are necessary for the different stainings, and a series of routine techniques are performed for the initial evaluation (histochemical and histoenzymatic techniques). Depending of the results of the routine panel, other more specific protocols can be applied to obtain additional information. HE, hematoxylin-eosin, PAS, periodic acid-Schiff, MG Trichrome, modified Gomori trichrome. Click here for file

Additional file 2: Table S1 Muscle biopsies and images used in this study. Information about the age and type of muscle is provided. Control muscles have been grouped according to their location (quadriceps, biceps or gastrocnemius muscle) and age (child or adult). Click here for file

Additional file 3: Figure S2 Block diagram of the steps followed in the segmentation process. The diagram includes images showing the output of the different steps. Click here for file

Additional file 4: Table S2 List of 82 characteristics analyzed and their values for each image. Values for the 82 characteristics of the 87 images used for the training step and the 15 other images tested. Av. = Average, s. d. = standard deviation. Characteristics 1 to 24 mimic features that the pathologist evaluates when analyzing a biopsy. The 24 characteristics can be classified into four groups: geometrically related to the size of fibers (1 to 6), geometrically related to the shape and orientation of fibers (7 to 14), geometrically related to the collagen content (15 and 16), and network characteristics (17 to 24). The network features capture information about the organization of the fibers. ***Area*:** Size (in pixels) of the fiber (A2). ***Major Axis*:** Length (in pixels) of the major axis of the ellipse that has the same normalized second central moments as the fiber. ***Minor Axis*:** Length (in pixels) of the minor axis of the ellipse that has the same normalized second central moments as the fiber. ***Relation Axis*:** Ratio between the major and minor axes of each fiber. ***Convex Hull*:** Proportion of the pixels in the convex hull that are also in the fiber. Computed as area of fiber/area of the convex hull. The convex hull is the smallest convex polygon that can contain the fiber. ***Angles*:** Angle (in degrees) between the x-axis (horizontal to the image) and the major axis of the ellipse that has the same second-moments as the fiber. ***Relation A1/A2*:** Ratio between the size (in pixels) of the ‘expanded fiber’ (A1) and the size of the fiber (A2, in pixels). ***Neighbors*:** Number of neighbor fibers of a fiber. Characteristics 25 to 38 are related to the value for a geometric characteristic of a node and the average value of its neighbors. A short description is given for characteristics 39 to 82: ***Strength***: Node strength is the sum of weights of links connected to the node, where the weight of links, in our case, is the distance in pixels between two fibers. ***Clustering coefficient***: The fraction of triangles around a node (equivalent to the fraction of a node’s neighbors that are neighbors of each other). ***Eccentricity:*** The shortest path length between a node and any other node. ***Betweenness centrality***: The fraction of all shortest paths in the network that contain a given node. Nodes with high values of betweenness centrality participate in a large number of shortest paths. ***Shortest path lengths***: The distance matrix containing lengths of shortest paths between all pairs of nodes. ***Radius:*** The minimum eccentricity. ***Diameter:*** The maximum eccentricity. ***Efficiency:*** The average inverse shortest path length in a network. ***Pearson***: The Pearson correlation reflects the degree of linear relationship between two variables (nodes and weight of links). ***Algebraic_connectivity***: The second smallest eigenvalue of the Laplacian (Laplacian: degree matrix minus the adjacency. Adjacency matrix: matrix with rows and columns labeled by graph nodes, with a 1 or 0 in position (v_i_, v_j_) according to whether v_i_ and v_j_ are adjacent or not). ***S_metric***: The sum of products of degrees across all edges. ***Assortativity***: A positive assortativity coefficient indicates that nodes tend to link to other nodes to the same or a similar degree. ***Density***: The fraction of present connections to possible connections. Connection weights are ignored in calculations. ***Transitivity***: The ratio of 'triangles to triplets' in the network (an alternative version of the clustering coefficient). ***Modularity:*** A statistic that quantifies the degree to which the network may be subdivided into such clearly delineated groups. Click here for file

Additional file 5: Figure S3 Scheme showing the approach proposed in this study. We present an example for the feature selection step using the artificial neuronal network (ANN) from the characteristics (yellow circle) and two categories (A and B) of images. Green and red squares represent two groups of images. The feature selection step provides the most discriminating characteristics for this comparison (orange circle) and the classification of the images into categories A and B. The blue squares represent new images. ANN classifies them into categories A and B using the selected characteristics. Principal component analysis (PCA) allows quantification of the degree of affection of the images used for the feature selection step, and also for the new images. This quantification is performed using the same selected characteristics. Click here for file
