# Supplementary figures and images for: Quantifiable diagnosis of muscular dystrophies and neurogenic atrophies through network analysis
Source: BMC Med. 2013 Mar 20;11:77. doi: 10.1186/1741-7015-11-77 (PMC3621542; doi:10.1186/1741-7015-11-77)

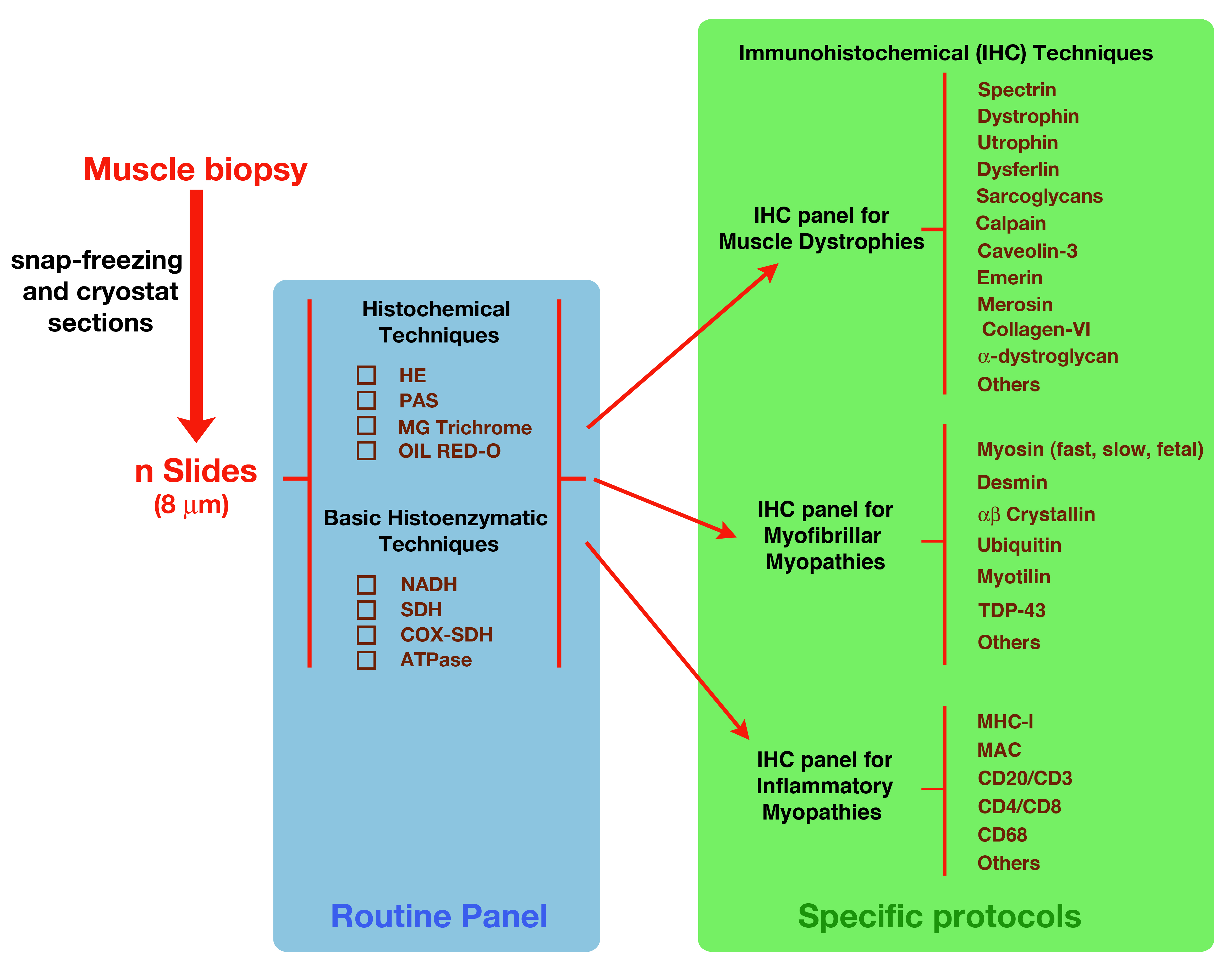

Supplement: Additional file 1: Figure S1 — Procedure for analysis of muscle biopsies in the neuropathology laboratory. Muscle biopsies are processed by cryostat. A large number of slides are necessary for the different stainings, and a series of routine techniques are performed for the initial evaluation (histochemical and histoenzymatic techniques). Depending of the results of the routine panel, other more specific protocols can be applied to obtain additional information. HE, hematoxylin-eosin, PAS, periodic acid-Schiff, MG Trichrome, modified Gomori trichrome. [file 1741-7015-11-77-S1.jpeg]

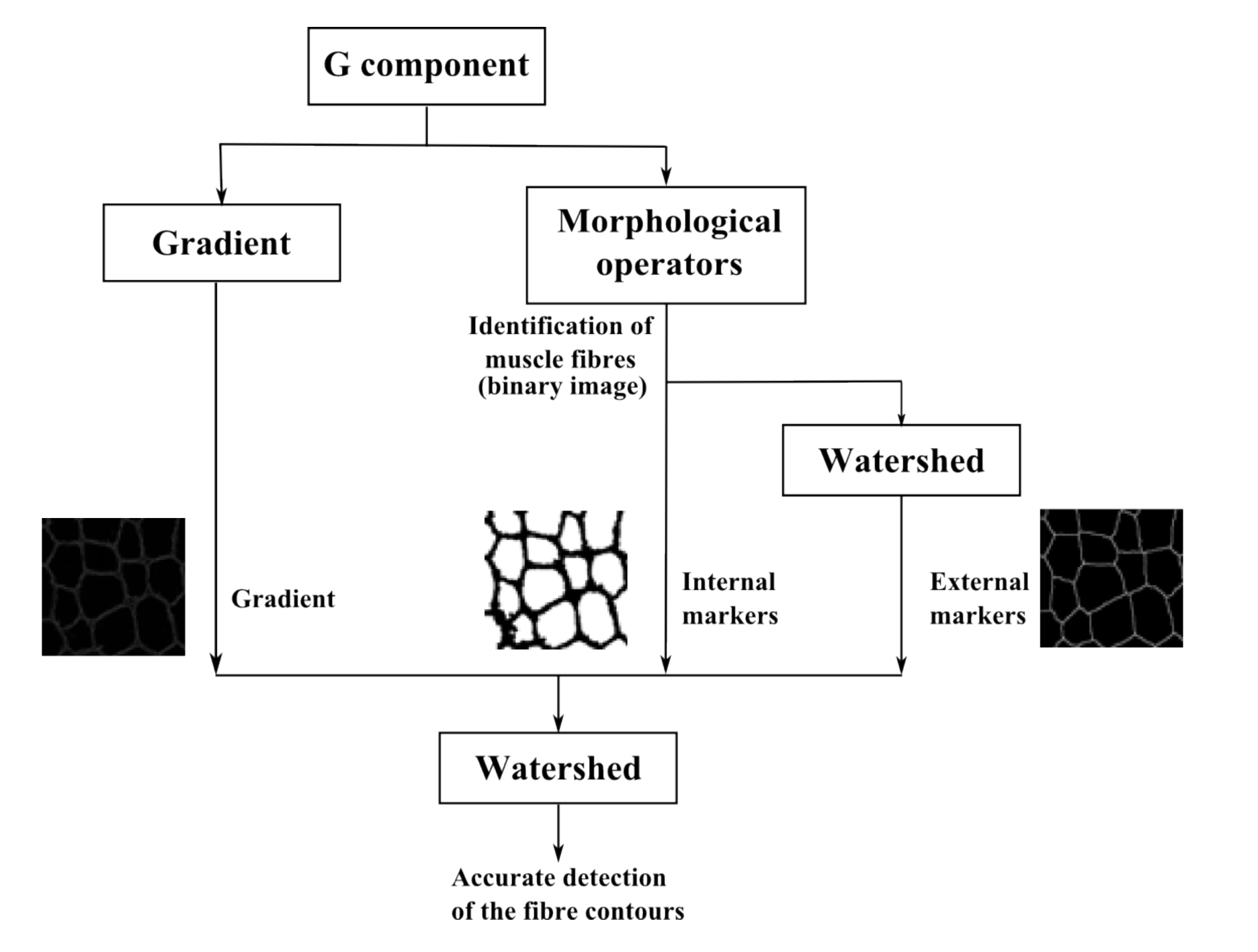

Supplement: Additional file 3: Figure S2 — Block diagram of the steps followed in the segmentation process. The diagram includes images showing the output of the different steps. [file 1741-7015-11-77-S3.jpeg]
